# Invertebrate and Vertebrate Class III Myosins Interact with MORN Repeat-Containing Adaptor Proteins

**DOI:** 10.1371/journal.pone.0122502

**Published:** 2015-03-30

**Authors:** Kirk L. Mecklenburg, Stephanie A. Freed, Manmeet Raval, Omar A. Quintero, Christopher M. Yengo, Joseph. E. O'Tousa

**Affiliations:** 1 Department of Biology, Indiana University South Bend, South Bend, Indiana, United States of America; 2 Department of Biological Sciences, University of Notre Dame, Notre Dame, Indiana, United States of America; 3 Department of Biology, University of Richmond, Richmond, Virginia, United States of America; 4 Department of Cellular and Molecular Physiology, Pennsylvania State University, College of Medicine, Hershey, Pennsylvania, United States of America; Texas A&M International University, UNITED STATES

## Abstract

In *Drosophila* photoreceptors, the NINAC-encoded myosin III is found in a complex with a small, MORN-repeat containing, protein Retinophilin (RTP). Expression of these two proteins in other cell types showed NINAC myosin III behavior is altered by RTP. NINAC deletion constructs were used to map the RTP binding site within the proximal tail domain of NINAC. In vertebrates, the RTP ortholog is MORN4. Co-precipitation experiments demonstrated that human MORN4 binds to human myosin IIIA (MYO3A). In COS7 cells, MORN4 and MYO3A, but not MORN4 and MYO3B, co-localize to actin rich filopodia extensions. Deletion analysis mapped the MORN4 binding to the proximal region of the MYO3A tail domain. MYO3A dependent MORN4 tip localization suggests that MYO3A functions as a motor that transports MORN4 to the filopodia tips and MORN4 may enhance MYO3A tip localization by tethering it to the plasma membrane at the protrusion tips. These results establish conserved features of the RTP/MORN4 family: they bind within the tail domain of myosin IIIs to control their behavior.

## Introduction

Myosins are a diverse family of actin binding proteins that function as molecular motors. Myosins have an amino-terminal motor domain, a short neck with one or more IQ repeats that serve as calmodulin or light chain binding regions, and a C-terminal tail containing protein-protein interacting motifs [1,2]. While much is known about the mechanism by which motors transduce chemical energy into movement, the mechanisms controlling cellular localization and regulation of cargo attachment are not well understood [3].

Class III myosins (MYO3) are novel in that they contain an amino-terminal kinase domain connected to the canonical myosin motor domain [4,5]. The founding MYO3 family member is NINAC, first identified as a *Drosophila* mutant showing an altered photoreceptor electrophysiological response to light [6]. A differentially spliced transcript leads to two NINAC proteins expressed in *Drosophila* photoreceptor cells [4]. The short form, NINACp132, is found predominantly in the cell body. The longer form NINACp174 localizes primarily to the rhabdomere, an actin rich microvillar structure that contains the molecular machinery for phototransduction [7]. Mutations that remove NINACp174 alter rhabdomere structure and the waveform of the light response.

Recently, two reports showed that a small 23 kD protein named retinophilin (RTP) interacts with NINACp174. RTP exhibits reversible light dependent phosphorylation suggesting it plays a regulatory role within the photoreceptor [8]. The stability of RTP is dependent on NINACp174 [8,9]. Further, RTP and NINAC co-localize to the rhabdomere and are identified as binding partners in co-immunoprecipitation studies [9]. RTP contains a motif known as a Membrane Occupation and Recognition Nexus (MORN). In other proteins, MORN repeats are responsible for membrane association and stabilization of protein complexes [10,11]

Vertebrates possess orthologs of both NINAC and RTP. While *Drosophila* retains the single MYO3 gene *ninaC*, expressed in photoreceptors, vertebrates possess two MYO3 genes MYO3A and MYO3B, expressed in multiple tissues with highest expression levels in the inner ear and the retina [5]. In vertebrate photoreceptors, MYO3A localizes to the calycal process, a protracted microvillar structure that cups the base of the light-sensitive outer segment [5]. Both MYO3A and MYO3B are found in stereocilia, the mechanosensing organelles responsible for hearing and balance [12,13]. Mutations in MYO3A lead to congenital deafness, while the photoreceptors are functional. MYO3B may compensate for defective MYO3A in photoreceptors and has been proposed to explain why only hearing is affected [14,15]. A single RTP ortholog, MORN4, has not been studied.

Here, we characterize the interactions of NINACp174 with RTP, and interactions of MYO3A with MORN4. The results show that both the *Drosophila* proteins and vertebrate proteins are found in protein complexes. The NINAC binding site for RTP was mapped to the putative control region within the carboxy tail. We found that the binding of RTP alters the *in vivo* behavior of NINACp174. In vertebrate MYO3A, but not MYO3B, a MORN4 binding site is also located within the carboxy tail. Our results demonstrate that RTP binds and regulates NINACp174 and MORN4 binds and enhances MYO3A tip localization, thus establishing conservation of RTP/MORN4 regulation of MYO3 motors in vertebrate and invertebrate systems.

## Materials and Methods

### Fly strains

The genotypes we generated were based on a *w* ^*1118*^ background. For histology, *cn bw* was introduced into the genetic background to remove eye pigment. Drivers for the UAS/GAL4 system were P{Rh1-GAL4} for transgene expression in photoreceptors R1-R6 [16], P{GawB}OK107 for expression in mushroom bodies [17], and P{GawB}AB1 GAL4 for expression in larval salivary glands [18]. *rtp* ^*1*^ was generated as described [8], and *ninaC* ^*P235*^ was obtained from William L. Pak (Purdue University).

### Molecular constructs

RTP-RFP was constructed as described previously [8]. The NINAC and MORN4 sequences were generated by PCR (Table 1). The NINAC sequences were cloned into pEntr/D-TOPO, and then recombined into pTGW using the Gateway system (Invitrogen). All constructs were sequence verified. Transgenic strains were generated following embryo injections (Bestgene) and genetic elements were combined by standard genetic methodology to create all genotypes described in this report. Human MORN4 was cut with EcoR1/BamH1 and ligated into a pmCherry expression vector [19]. For generating GST-MORN4 recombinant protein, MORN4 was inserted into the BamHI and NotI restriction sites of pGEX-1 vector (gifted by Bechara Kachar, NIH). The GFP-tagged expression constructs MYO3AΔK and 3A Tail FL constructs were developed previously [12,20] for cell biological analysis. GFP-MYO3AΔK construct lacks the kinase domain (aa340-1616 of NM_017433.4), whereas, the GFP-MYO3A Tail construct lacks the kinase and motor domains (aa1133-1616 of NM_017433.4). GFP-MYO3A Tail deletion constructs were generated by performing site-directed mutagenesis on the GFP-MYO3AΔK construct and the primers listed in Table 1, thus they also lack the kinase domain. The GFP-MYO3AΔK, 34 tail deletion construct was described previously [13]. Full-length human MYO3B (NM_138995) construct was developed in our laboratory from the template DNA gifted by Bechara Kachar, NIH. MYO3B full length was inserted into the BamHI and NotI restriction sites of pEGFP vector. The GFP-MYO3BΔK.3THDII chimeric construct encoding for human MYO3B with its N-terminal kinase domain deleted (aa 345–1314 of NM_138995.4) and fused to 63 C-terminal amino acids (aa 1554–1616 of NM_017433.4) human MYO3A sequence corresponding to its 3THDII domain was generated by two step megaprimer PCR method. The 3THDII domain sequence containing megaprimer was PCR cloned using GFP-MYO3AΔK construct as the template in the first step. The megaprimer was then used for the second step PCR using GFP-MYO3BΔK construct as the template to insert 3THDII at the C-terminal end of the MYO3BΔK sequence.

**Table 1:** List of plasmid constructs and associated primers.

Plasmid (aa deleted)	Vector	Gene/Species (Accession no.)	Primers used, Forward (F) and Reverse (R)
NINAC-FL	pEntr/D-TOPO, then pTGW	NinaC/Drosophila (NM_078779)	F:CACCATGATGTATTTACCGTAC,R:CACTAGTTTTAGATATCGACGG
NINAC-TAIL	pEntr/D-TOPO, then pTGW	NinaC/Drosophila (NM_078779)	F:CACCATGGTGCAGTCCATGATGCGAG,R:CACTAGTTTTAGATATCGACGG
TD1	pEntr/D-TOPO, then pTGW	NinaC/Drosophila (NM_078779)	F:CACCATGGTGCAGTCCATGATGCGAG,R:TTACTGATTGTAGATATTGTCGCTC
TD2	pEntr/D-TOPO, then pTGW	NinaC/Drosophila (NM_078779)	F:CACCATGAATAAATGTACTCGGGCTG,R:TTACAGCATAGCCTTGAAG
TD3	pEntr/D-TOPO, then pTGW	NinaC/Drosophila (NM_078779)	F:CACCATGCAGGGCTACTTCCGAGA,R:CACTAGTTTTAGATATCGACGG
GFP-M3A ΔK,30 (1134–1431)	pEGFP-N1	Myo3A/Human (NM_017433)	F:CGACTACAAGAAAAACTTTGAAAATACAGGATATCAAAAGTTATCTGAAGAATATTTCAT,R:ATGAAATATTCTTCAGATAACTTTGATATCCTT
GFP-M3A ΔK,31 (1432–1479)	pEGFP-N1	Myo3A/Human (NM_017433)	F:TTTGGCAATTTTTTCAAAACAGGGTGTCTGTAAAGGAGAGGAG,R:CTCCTCTCCTTTACAGACACCCTGTTTTGAAAAAATTGCCAAA
GFP-M3A ΔK,32 (1480–1515)	pEGFP-N1	Myo3A/Human (NM_017433)	F:CAGCAGTGCCTCTCAGGTAAGTCAATCCAAGAAG,R:CTTCTTGGATTGACTTACCTGAGAGGCACTGCTG
GFP-M3A ΔK,33 (1516–1528)	pEGFP-N1	Myo3A/Human (NM_017433)	F:CTGAAGACTCCACATACTATTATCTACTTCATAGTCAGGGAAAATTATTAG,R:CTAATAATTTTCCCTACTATGAAGTAGATAATAGTATGTGAGTCTTCAG
GFP-M3A ΔK,34 (1529–1576)	pEGFP-N1	Myo3A/Human (NM_017433)	F, R: see Schneider et al., 2006
GFP-M3B ΔK.3THDII	pEGFP-N1	Myo3B/Human (NM_138995)	F:CCTCAAAAGGAGACCTTTTGCTCAACATTAACATAGCCCTAGTTTAAGAGAACGAAAC,R:TATCTAGATGCATGCTCGAGCGGCCGCTAGGACTGCTGGACGAGGCG
GST-MORN4	pGEX-1	MORN4/Human (NM_001098831)	F:GCGGATCCGGCATGACCCTGACAAAAG,R:GCGGCCGCTCAGGCAGTGAGATTTC
mCherry-MORN4	pmCherry	MORN4/Human (NM_001098831)	F:AAAGAATTCTATGACCCTGACAAAAGGTTCCT,R:TTTGGATCCTCAGGCAGTGAGATTTCTGGCTGACTTG

### Histology

For *Drosophila* tissue sections, fly heads were fixed in 4% paraformaldehyde/5% sucrose. The following day, the heads were washed 3X for 10 minutes in 5% sucrose/PBS, and incubated overnight at 4°C. The solution was removed and heads were incubated in 30% sucrose/ PBS overnight at 4°C. This solution was then removed and heads were placed in 30% sucrose/1X PBS:Tissue-Tek OCT for 4 hours at room temperature. 10 um sections were then cut on a Microm HM525 cryostat, and the samples were washed in PBS for 20 minutes. Larval brains and salivary glands were obtained from crawling 3^rd^ instars by dissection in PBS. Brains and salivary glands were fixed in 4% paraformaldehyde/PBS for 10 minutes, and transferred to PBS. The tissues were then mounted with Vectashield or Vectashield plus DAPI (Vector Laboratories).

Fluorescence was detected with a Leica DM5000B microscope equipped with a Leica DFC300 FX camera and Fluotar 40X/0.75 objective. Confocal images were obtained on a Bio-Rad MRC 1024 microscope equipped with a Nikon Diaphot 200 and Plan-Apochromomat 60X, NA 1.40 objective. Both digital photomicrographs and confocal images were adjusted using Adobe Photoshop CS3 software for scaling and uniform contrast and brightness.

### Protein blots

Heads were removed from newly eclosed animals and homogenized in 25 μl solubilization buffer, (60 mM Tris-HCl, pH 6.8, 20%SDS, 0.0004% bromophenol blue, 10% ß-mercaptoethanol, 20% glycerol) and diluted with 25 μl H_2_O. The homogenate was incubated 1 hour at 37°C and loaded onto a NuPAGE 4–12% Bis-Tris Gel (Novex) and size fractionated. Proteins were electroblotted to Immobilon-P polyvinylidene difluoride membranes (Millipore) using a Mini Trans-Blot electrophoretic transfer cell (Bio-Rad). Membranes were blocked (5% BioRad Blotting-Grade blocker in PBS) for 1 hr, and primary anti-RTP antibody 1:2000 in blocking buffer was added and incubated overnight at 4°C. Membranes were washed 3X for 5 minutes in TBST pH 7.4. Secondary antibody, goat anti-rabbit IgG conjugated to horseradish peroxidase (GE Healthcare) in blocking buffer (1:3000) was added for 1 hour, then washed 3X for 5 minutes pH 7.4. Detection was with ECL HRP chemiluminescence as specified by the manufacturer (Invitrogen Novex). RTP antibody was generated against the peptide GFPRNEGFFQDCRFMR. Peptide preparation, rabbit immunization, and antibody affinity purification was carried out by Biomatik (Cambridge, ON).

### Protein Interaction Analysis

Recombinant GST-MORN4 protein was expressed in Rosetta cells and purified from bacterial lysates by using Immobilized Glutathione-agarose beads (Thermo Scientific). GFP- 3ATail FL fusion protein containing COS7 lysates were prepared as described previously [20]. Briefly, COS7 cells were transfected with various constructs and cell lysates containing fusion proteins were extracted from COS7 cells after 24-hr of transfection by brief sonication in ice cold lysis buffer (CLB: 5mM DTT, 150mM NaCl, 1% Triton X -100, 50mM Tris pH 7.4, 2mM EDTA, 1mM PMSF, Aprotenin, Leupeptin) and 20 min ultracentrifugation at 58,000 rpm using a Beckman Optima MAX ultracentrifuge with a TLA 120.2 rotor. To test for MYO3A interactions, the GST-MORN4 or GST alone was bound to the Glutathione agarose beads for 1hr at 4°C followed by incubation with GFP-tagged MYO3A tail fragments in CLB for 2hr. The agarose beads were then washed 3 times with 1X cPBS and the final pellet was resuspended in SDS-PAGE buffer. The co-precipitates were separated on NuPAGE Bis-Tris 4–12% gels and transferred to nitrocellulose membrane for analysis by western blotting using rabbit polyclonal anti-GST (CALBIOCHEM) and anti-GFP antibodies (Invitrogen).

### Cell culture and transient transfection

COS7 cell cultures were propagated in DMEM (Invitrogen) supplemented with 4mM L-glutamine, 4.5g/L D-Glucose, 1mM Sodium Pyruvate, 10% fetal bovine serum, and 100 units of penicillin-streptomycin. Cultured cells were maintained at 37°C with 5% CO_2_ in air. For transfection, 30,000–40,000 cells were plated on acid washed 22mm square glass coverslips and allowed to adhere overnight. Cells were transiently transfected using FUGENE HD transfection reagent (Promega) and imaged after ~20–30 hours. For transient transfection, 0.4μg of the GFP-MYO3 plasmids and/or 0.1μg of mchr-MORN4 plasmid (0.5μg total for co-transfections) were used.

### Live- and fixed-cell imaging of COS-7 cells and data analysis

Fixed cell imaging of COS-7 cells was used to visualize localization of actin, GFP-MYO3AΔK, and/or mchr-MORN4. Cells were fixed 24 hours after transient transfection. Samples were fixed for 20 min in 4% paraformaldehyde in phosphate buffered saline (PBS), permeabilized for 30 min in 0.5% Triton X-100 in PBS and then counterstained with 2ml of 0.0001 units/μl Alexa Fluor-647 Phalloidin (Molecular Probes) to label actin. Fluorescence images were obtained using Olympus IX71 with 100X 1.40 oil immersion objective and deconvolved using DeltaVison SoftWoRx software (Applied Precision). Live cell imaging of transfected cells was used for quantification of tip/cell body ratio. Coverslips were placed in Rose chambers filled with Opti-MEM without phenol red and supplemented with 5% fetal bovine serum and 100 units of Penicillin-streptomycin. Images were acquired using a Nikon TE2000-PFS fluorescence microscope with a 60x/1.4 N.A. phase objective. NIS-Elements AR (Nikon) and ImageJ were used to analyze the images and to quantify tip to cell body intensity ratio, as described previously [19]. Data were analyzed by one-way analysis of variance (ANOVA) with subsequent Tukey post-hoc analysis using GraphPad Prism 6 software. Data are expressed as means ± SE to establish significant differences between two different conditions.

## Results

### The RTP/NINAC interaction motif maps to the NINAC proximal tail

**Fig 1:**
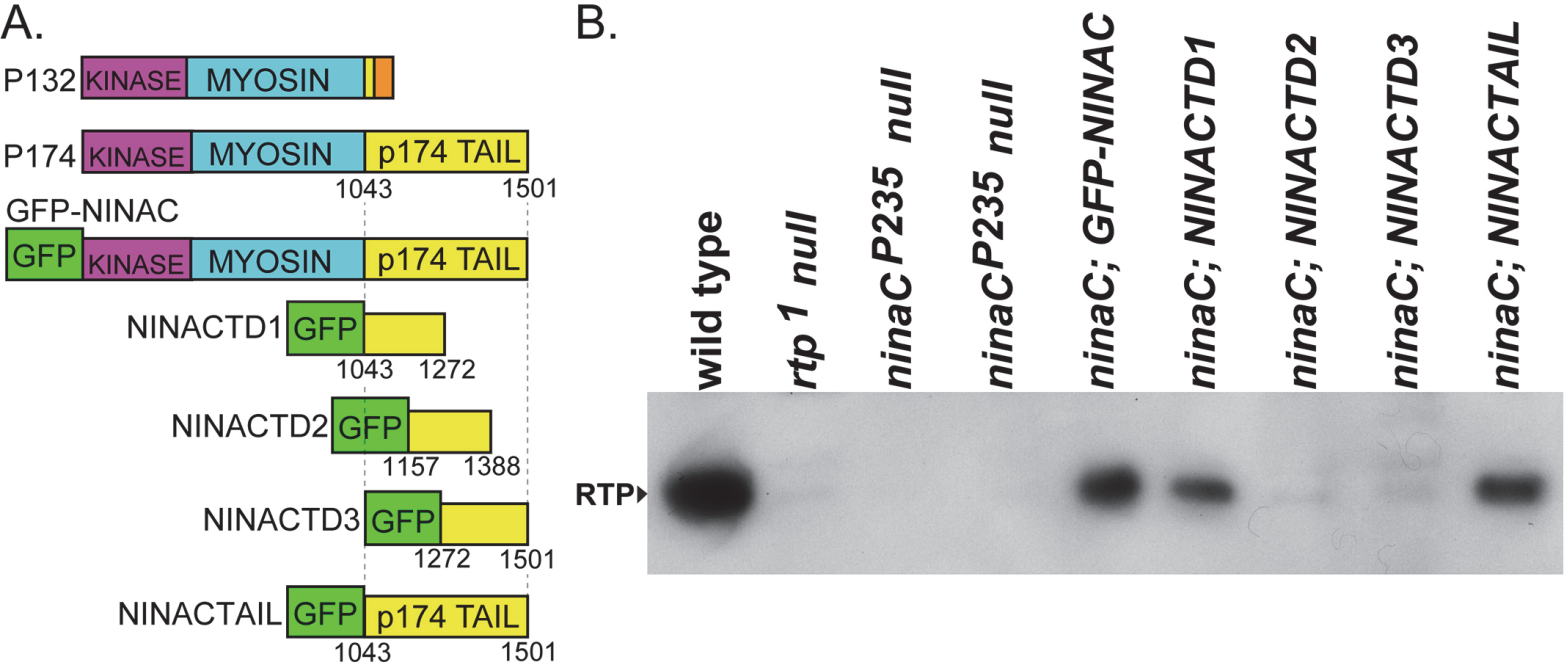
The proximal tail region of NINAC mediates RTP stabilization. (A) The two upper left entries are a diagram of the P132 and P174 NINAC proteins, indicating the location of the shared kinase and myosin head domains and the distinctive tail domains. In orange is the region unique to P132. The GFP-NINAC construct creates GFP fused to the N-terminus of the full length NINAC protein (not shown). The GFP-NINAC fusion proteins used in this study are diagrammed below P132 and P174 NINAC. These constructs contain GFP fused at the N-terminus of NINACTD1 (= Tail Domain 1, etc.), NINACTD2, NINACTD3 and NINACTAIL proteins. Numbers indicate the first and last NINACp174 amino acid present in these fusion proteins. (B) Protein blot showing the capability of the GFP-NINAC, NINACTD1, and NINACTAIL proteins, but not NINACTD2 and NINACTD3 proteins, to support RTP expression within photoreceptors. The four lanes on left are control genotypes.

Previous results showed that the longer NINACp174 isoform supports RTP stability, but the shorter NINACp132 does not, suggesting that this property is conferred by a NINAC region unique to NINACp174. The domain structure of the two NINAC isoforms is shown in the top two entries of Fig 1A. To confirm and map the RTP binding site, we created *Drosophila* transformants carrying four different GFP-NINACp174 tail regions as diagrammed in Fig 1A. The first three are an overlapping set of constructs with 50% of the p174 tail sequences and named NINACTD (Tail Domain) TD1, TD2, and TD3, the fourth is a transgene with the entire p174 tail (NINACTAIL). Each of these constructs were placed under UAS promoter control, and expression in the R1-6 photoreceptor cells driven by the Rh1-GAL4 driver element [18]. Expression of all transgenes was confirmed by the presence of GFP within the adult eye.

To determine if the NINAC tail constructs were capable of supporting RTP stability, we expressed each in *ninaC* ^*P235*^ null mutants and examined RTP expression. Fig 1B shows a protein blot assessing RTP levels in controls and experimental genotypes. RTP is readily detected as a 23 kD protein in wild type but is absent in both the *rtp1* and *ninaC* ^*P235*^ null mutants. The results show animals with full length NINACTAIL and NINACTD1 retain RTP, but NINACTD2 and NINACTD3 do not. Thus the NINACTD1 region of NINACp174 is necessary and sufficient for RTP stability.

**Fig 2:**
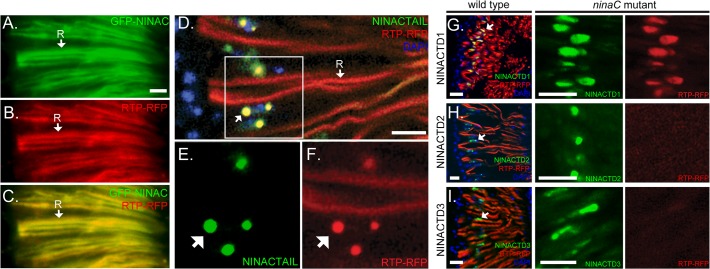
The proximal tail region of NINAC mediates RTP colocalization. (A-C) Images obtained from fluorescent microscopy show that GFP-NINAC and RTP-RFP both localize to the photoreceptors’ rhabdomeric membranes (labeled R, arrow). (D-F) Confocal retina images of RTP-RFP and GFP-NINACTAIL localization with native NINAC expression. The boxed region in (D) is magnified for GFP-NINAC labeled in (E) and for RTP-RFP labeled in (F). One rhabdomere, labeled by RTP-RFP, is identified as “R” in D. The arrows in (D-F) identify cytoplasmic structures containing both RTP and NINACTAIL. (G-I) The GFP tagged tail constructs TD1, TD2 and TD3 expressed in cells with native NINAC, are found in proximity to the photoreceptor R1-6 nuclei (left column, DAPI staining labeled blue). RTP is found in the rhabdomere, and colocalizes only with the NINACTD1 construct (yellow). In middle and right columns, the GFP-tagged tail constructs TD1, TD2 and TD3 are coexpressed with RTP-RFP in a *ninaC* ^*P235*^ null mutant background. RTP is found associated with the cytoplasmic structures containing NINACTD1, but is absent from cytoplasmic structures containing NINACTD2 or NINACTD3. Scale bars, 10 μm.

If RTP is stabilized by binding to NINAC, we reasoned that RTP would co-localize with each of the NINAC constructs that support RTP expression. To assess this, we constructed genotypes with each GFP-tagged NINAC construct and RTP-RFP. The full length GFP-NINACp174, containing both the kinase and myosin domains, and RTP-RFP are found at the expected rhabdomeric location (labeled R, Fig 2A–2C). In contrast, the NINACTAIL construct is predominantly localized to spherical structures located distally near the photoreceptor nuclei (Fig 2D–2E, nuclei labeled in blue, arrow identifies one spherical structure). RTP presence and location was monitored by detection of RTP-RFP. RTP is found in the rhabdomeres, as expected from association with native NINAC, but also in the spherical structures due to association with NINACTAIL (Fig 2D–2F). Similar experiments with NINACTD1, NINACTD2 and NINACTD3 are shown in Fig 2G–2I, left panels. RTP-RFP exhibited colocalization with NINACTD1 (Fig 2G, arrow shows site labeled yellow due to combined red and green signals) but not with NINACTD2 or NINACTD3 (arrows show perinuclear sites labeled only as green).

This analysis was extended by expression of RTP-RFP with NINACTD1, NINACTD2 orNINACTD3 in a NINAC null mutant. This eliminated RTP association with native NINAC in the rhabdomere. These results, (Fig 2G–2I, middle and right panels) showed that the NINACTD2 and NINACTD3 domains fail to express detectable RTP-RFP (Fig 2H and 2I). In contrast, photoreceptors possessing NINACTD1 show RTP-RFP localizing within the NINAC GFP-labeled structures in the distal photoreceptor (Fig 2G). Thus, these results show a protein motif located within the NINACTD1 region is responsible for RTP stability and NINAC co-localization.

### Ectopic expression shows the RTP/ NINAC complex forms outside the photoreceptor environment

**Fig 3:**
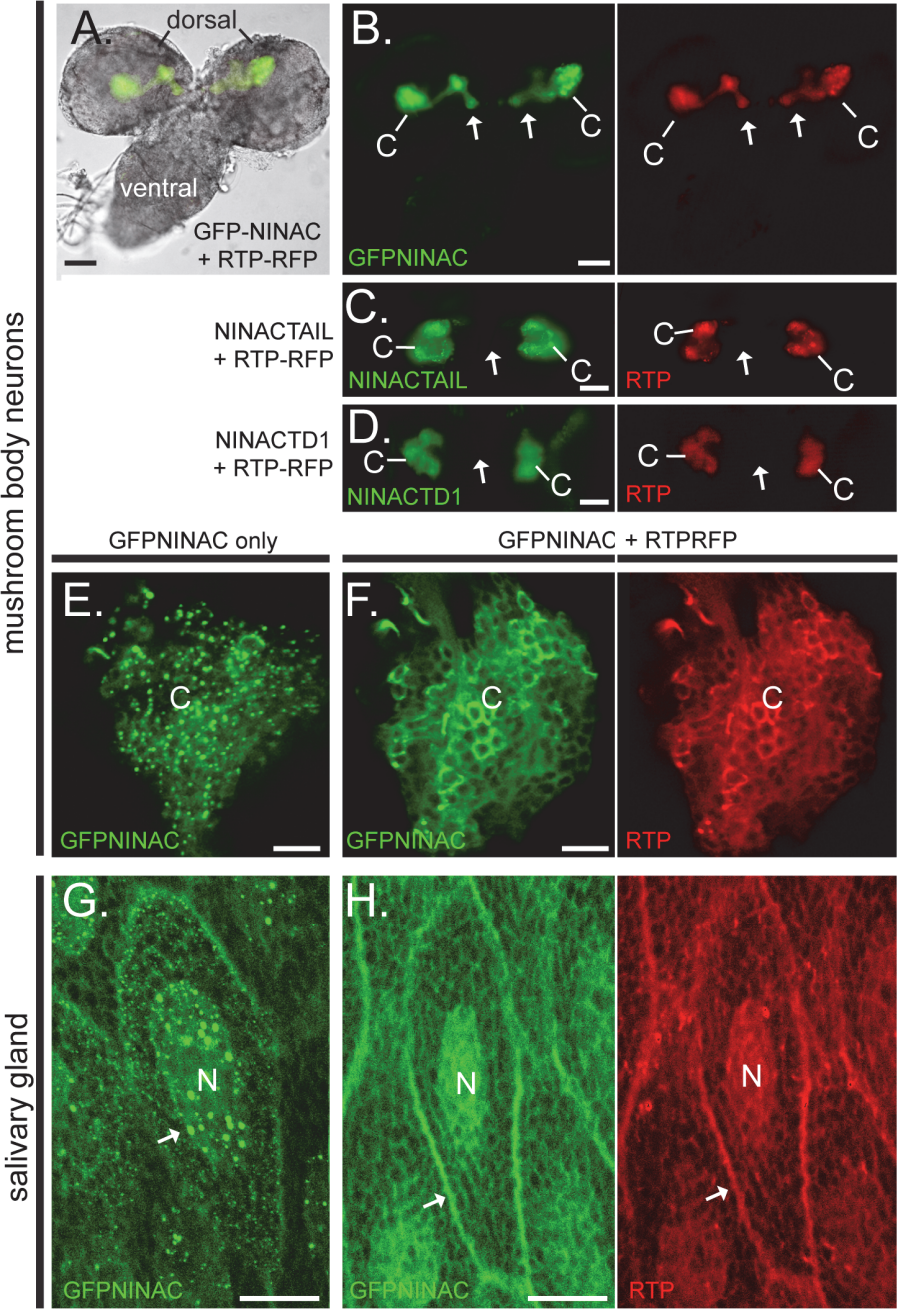
RTP and NINAC interact in multiple *Drosophila* cell types. (A-F) The GFP-NINAC tail domain constructs were expressed with and without RTP-RFP in larval mushroom body neurons. (A) A bright field image of larval brain, overlaid with fluorescence images specifying GFP-NINAC and RTP-RFP location is shown. The dorsal and ventral brain regions are labeled. (B) Left and right panels are the fluorescent images from the larval brain shown in (A) detecting the location of GFP-NINAC and RTP-RFP respectively when both are expressed simultaneously. GFP-NINAC and RTP-RFP are colocalized within the Kenyon cell bodies (labeled C) as well as projections through the peduncle and into the neuronal lobes (arrows). (C-D) When RTP-RFP is expressed with NINACTAIL and—NINACTD1, both GFP-NINAC and RTP-RFP are localized only within the Kenyon cell bodies and not within the peduncle structures (arrow). (E,F) 1.5 μm confocal optical sections of the Kenyon cells (C) of the mushroom bodies expressing GFP-NINAC only (E) or GFP-NINAC and RTP-RFP (F). In the absence of RTP, the GFP-NINAC protein is found within punctate structures, while the presence of RTP-RFP prevents formation of GFP-NINAC puncta. (G,H) 1.5 μm confocal optical section showing GFP-NINAC only (G) or GFP-NINAC and RTP-RFP (H) localization in larval salivary gland cells. GFP-NINAC is found within punctate structures within the nucleus (labeled N), while expression of RTP-RFP prevents formation of these GFP-NINAC punctate structures and the two proteins are colocalized within the cytoplasm, likely associated with internal membranes (arrow). Scale bars, A-D: 50 μm, E,F: 10μm, G,H: 20 μm.

Because endogenous NINAC localizes to the rhabdomere and is thus influenced by the photoreceptor cell environment, we sought to characterize the behavior of NINAC and RTP in the absence of photoreceptor morphological structures and photoreceptor-specific proteins. We first used the P{GawB}OK107 GAL4 driver [17] to express GFP-NINAC and RTP-RFP proteins in the larval mushroom body neurons. Fig 3A shows the larval brain consisting of two dorsal spheres connected by the ventral region. Within the dorsal spheres, the mushroom bodies are identified by the expression of GFP-NINAC and RTP-RFP. These two co-expressed proteins are located within the Kenyon cell bodies (C) and within the axonal peduncle projections (arrows). GFP-NINAC expressed alone was readily detected in the mushroom body neurons; however, RTP-RFP expressed alone was not detected (data not shown). We then examined the behavior of RTP with NINAC tail domains. As in photoreceptors, NINACTD1 and NINACTAIL expression stabilizes RTP-RFP in the mushroom body cortex (Fig 2C and 2D) while NINACTD2 and NINACTD3 do not (data not shown). Thus the behavior of RTP with different NINAC tail domains in the mushroom body neurons confirms that RTP stabilization is dependent on the NINACTD1 region of the NINACp174 tail. Further, it shows that RTP stabilization is not dependent on other photoreceptor specific components.

### RTP expression prevents the formation of RTP/NINAC puncta

The ability to express NINAC/RTP in the mushroom bodies allowed us to examine details of their interactions. A magnified view of the mushroom body Kenyon cell region shows that NINAC expressed without RTP is found in discrete puncta (Fig 3E). Co-expression of NINAC with RTP broadened this distribution and prevented NINAC puncta formation (Fig 3F). We also examined NINAC/RTP interactions in large, non-neuronal cells by driving transgene expression in the larval salivary glands with P{GawB}AB1 GAL4. When GFP-NINAC was expressed in the absence of RTP, GFP-NINAC localized to puncta within the cell nuclei (arrow, Fig 3G) and cytoplasm. As the case for photoreceptors and mushroom body neurons, RTP was not present in the absence of NINAC (data not shown). GFP-NINAC, in the presence of RTP-RFP, did not accumulate within the cell nucleus but instead distributed uniformly on internal and plasma membranes (arrow, Fig 3H). Thus, ectopic expression of NINAC in two different cell types confirmed that RTP is stabilized in the presence of NINAC, and that RTP prevents formation of RTP/NINAC puncta.

### Vertebrate NINAC and RTP orthologs, MYO3A and MORN4, are binding partners

**Fig 4:**
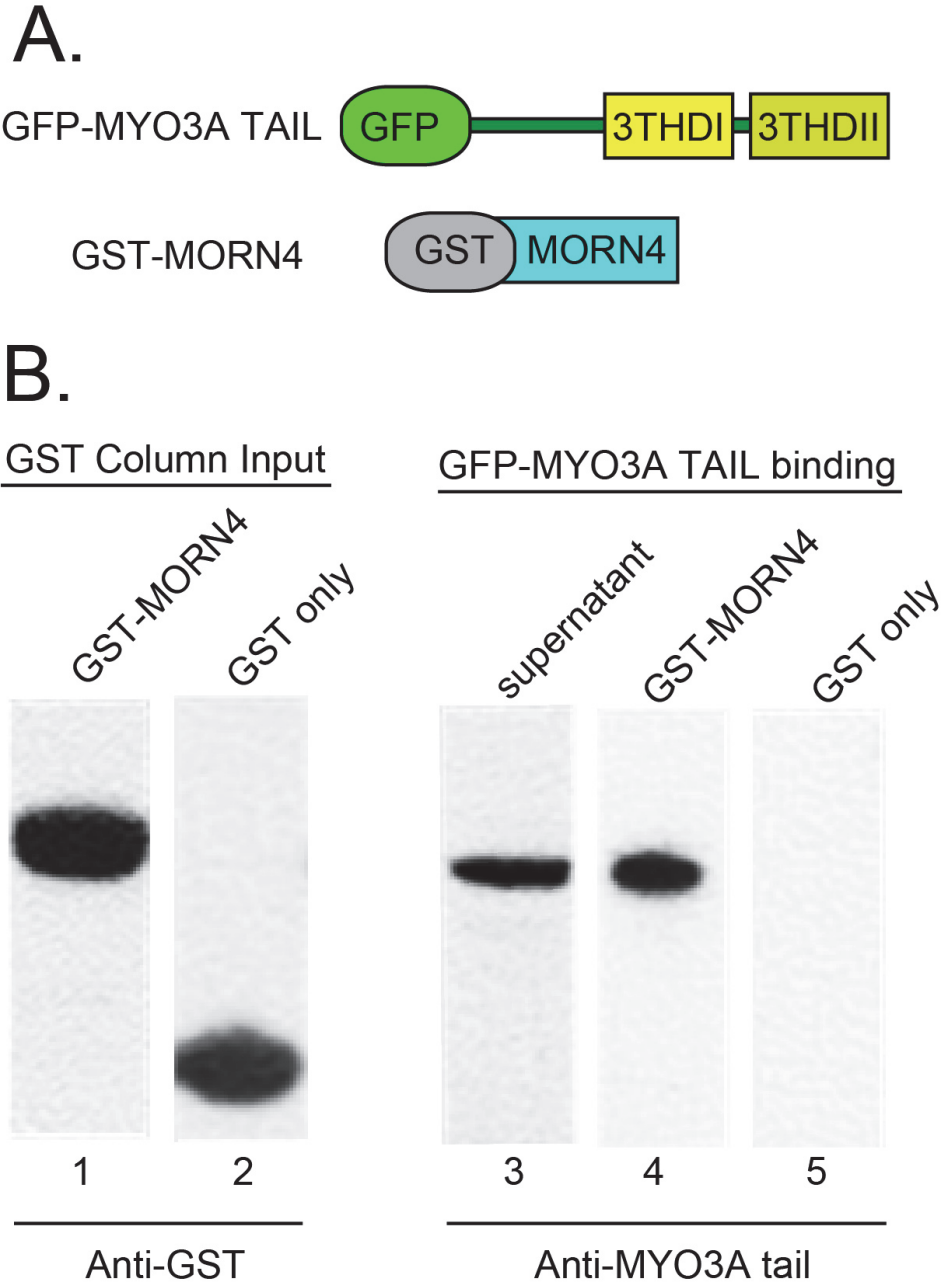
MYO3A tail binding MORN4 demonstrated by GST-pulldown assays. (A) Schematic map of the constructs used for analysis of the MYO3A and MORN4 protein interaction. (B) Protein blots show GST-MORN4 (lane 1) and GST-only (lane 2) proteins were expressed and purified from bacterial lysates. Protein blots confirmed that lysates from transfected COS-7 cells express the GFP-MYO3A tail FL protein (lane 3), and this protein could be pulled out of the lysate with GST-MORN4-glutathione-agarose beads (lane 4) but not with GST- only glutathione-agarose beads (lane 5). Anti-MYO3A tail tip primary antibodies (PB638) were used to detect the GFP-MYO3A tail FL protein in the lysate and pull down fractions.

Mammals possess two NINAC homologs, MYO3A and MYO3B, and the RTP-like protein MORN4. To determine if the MYO3A and MORN4 proteins recapitulate the NINACp174/RTP interaction, we examined if they were binding partners, and if they colocalized when expressed in COS-7 cells. To purify MORN4 protein for binding studies we created a GST-MORN4 plasmid, and to express the putative MORN4 binding partner, we used a GFP-MYO3A Tail FL construct [12]. These proteins are diagrammed in Fig 4A. The GST-MORN4 protein, and the control GST only protein were purified by binding to glutathione-agarose beads (Fig 4B, lanes 1, 2). MYO3A full-length tail protein was expressed in COS-7 cells (Fig 4B, lane 3). This MYO3A full length tail protein bound to the GST-MORN4 loaded agarose beads (Fig 4B, lane 4) but not the GST only loaded agarose beads (Fig 4B, lane 5). These results establish that the MYO3A tail domain contains a MORN4-binding motif.

### Fluorescently tagged MYO3A and MORN4 colocalizes at the filopodia tips of transfected COS7 cells

**Fig 5:**
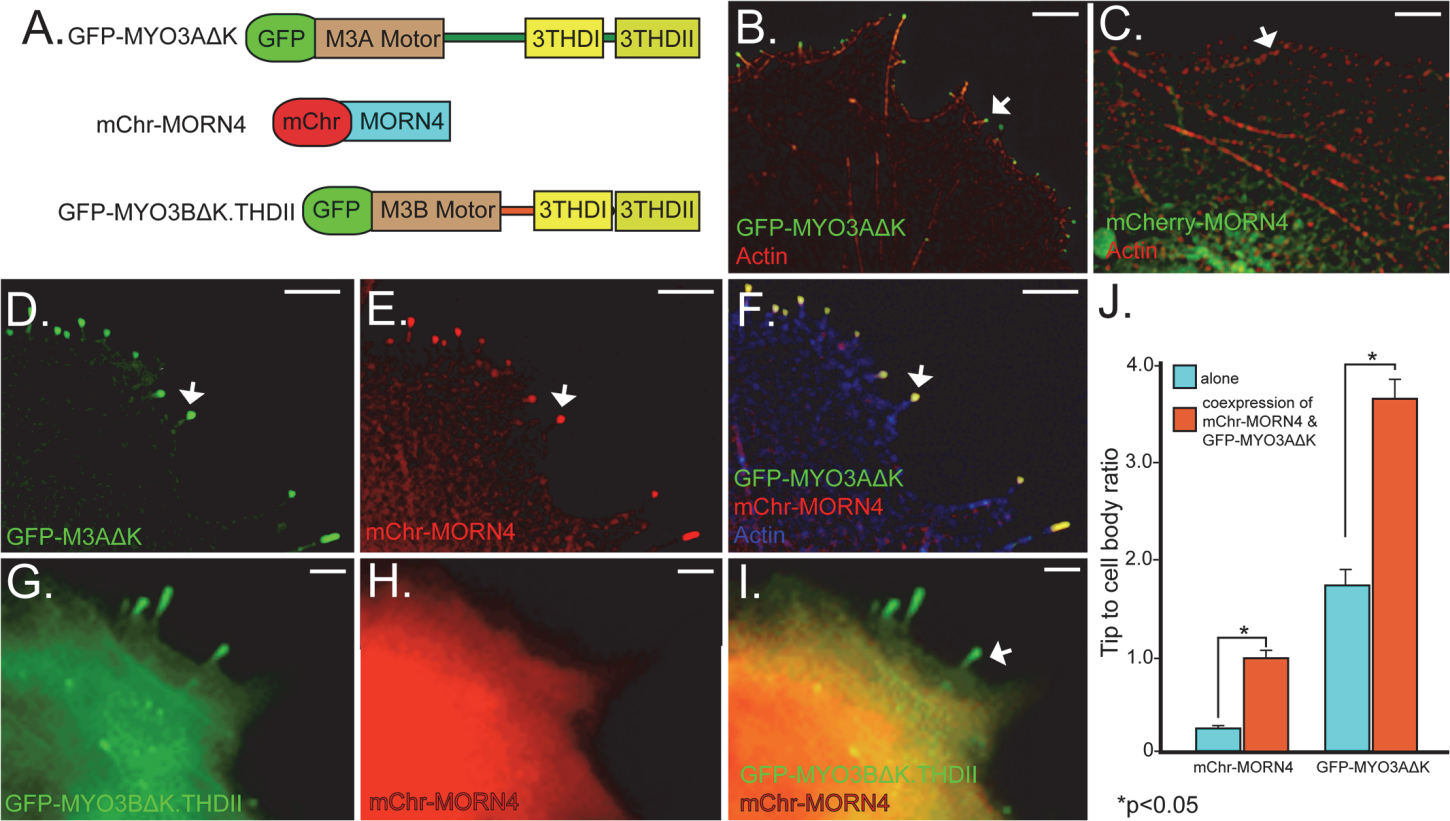
mChr-MORN4 co-expression with GFP-MYO3AΔK, but not GFP-MYO3BΔK.3THDII, in COS7 cells promotes filopodial localization of mChr-MORN4. (A) Schematic map of the constructs used for transfections. The Myosin constructs are tagged with GFP, and the MORN4 construct with RFP. The MYO3AΔK and MYO3BΔK constructs consist of the motor and the tail domains but they are deleted for kinase domain to enhance tip localization. The MYO3A 3THDII domain has been added to the MYO3BΔK.THDII construct to promote actin binding. (B) COS7 cells transfected with the GFP-MYO3AΔK (green) shows GFP-MYO3AΔK localization to filopodial tips at the cell membrane (arrow). (C) When expressed in absence of GFP-MYO3AΔK, mChr-MORN4 protein (green) is found within the cell body and not concentrated at the filopodial tip at the cell membrane (arrow). (D-F) Coexpression of GFP-MYO3AΔK (green, D, F) with mChr-MORN4 (Red, E, F) results in filopodial tip localization of mChr-MORN4 (arrow). In panel (F), actin is shown in blue. In (D-F), the arrow identifies one filopodial tip with mChr-MORN4 and GFP-MYO3AΔK colocalization. GFP-MYO3BΔK.THDII fails to localize mChr-MORN4 to the filopodial tips in COS7 cells. (G-I) Coexpression of GFP-MYO3BΔK.THDII (G, I, green) with mChr-MORN4 (H, I, red) fails to localize mChr-MORN4 to filopodial tips. The GFP-MYO3BΔK.THDII protein (green) is localized to filopodial tips (arrow in I). (J) Tip to cell body ratio measurements of GFP-MYO3AΔK and mChr-MORN4 in COS7 cells under different conditions. mChr-MORN4 tip localization was significantly higher in COS7 cells co-expressing mChr-MORN4 and GFP-MYO3AΔK (n = 317 filopodia from 24 cells) as compared to mChr-MORN4 alone (P<0.0001; T-test) (n = 175 filopodia from 20 cells). Similarly, COS7 cells co-expressing GFP-MYO3AΔK and mChr-MORN4 show significantly higher GFP-MYO3AΔK at the filopodial tips as compared to GFP-MYO3AΔK alone (p<0.0001) (n = 206 filopodia from 19 cells). Scale bars, 5 μm.

We investigated the cellular interactions between MYO3A and MORN4 by expressing fluorescently tagged versions of both proteins (Fig 5A) in COS-7 cells. We and others have successfully used COS7 cells in previous studies to perform cell biological and biochemical assays for investigating MYO3A function, regulation and its interaction with its known cargo protein ESPN1 [12,13,19,20]. COS7 cells are an adherent cell-line that are easy to culture and transfect which makes them an ideal choice for live cell imaging of fluorescently tagged proteins. COS7 cells have the property to form actin based filopodial structures similar to vertebrate actin rich stereocilia and calycal processes, where MYO3A is found to be expressed endogenously. We used the GFP-MYO3AΔK construct lacking the kinase domain because the kinase domain of MYO3 down-regulates myosin motor activity. The increased activity of the myosin motor provides for dynamic localization to filopodia tips (Fig 5B *)* as expected from prior studies [13,14,19,20]. In the absence of GFP-MYO3AΔK, mChr-MORN4 is broadly distributed, being found within the nucleus and cytoplasm but absent from the filopodia or other actin-based structures (Fig 5C and 5H). We reasoned that in the presence of GFP-MYO3AΔK, mChr-MORN4 would be brought to the filopodial tips if these two proteins are binding partners. Consistent with this expectation, when mChr-MORN4 was expressed in the presence of GFP-MYO3AΔK, mChr-MORN4 colocalized with the GFP-MYO3AΔK protein at the filopodia tips, (Fig 5D–5F).

### MORN4 specifically binds MYO3A and enhances its tip localization in COS7 cells

To further define the binding specificity of MORN4 with MYO3A, we examined if the closely related MYO3B could also serve as a binding template. MYO3A and MYO3B contain distinct tail regions, though retain sequence identity within tail homology domain I (THDI) [12,20]. Since MYO3B lacks the actin binding motif in tail homology domain II (THDII), MYO3B does not localize to the filopodia tips of COS-7 cells. However a chimera of MYO3BΔK containing THDII at the C-terminus (GFP-MYO3BΔK.THDII) will localize to the filopodia tips (Fig 5G). We find that the GFP-MYO3BΔK.THDII construct does not direct MORN4 to the filopodia tips when these two proteins are co-transfected (Fig 5H and 5I).

We quantified the distribution of mChr-MORN4, GFP-MYO3AΔK and GFP-MYO3BΔK.THDII in COS7 cells by examining the filopodia tip/cell body ratio as done previously [19]. We observed a statistically significant 3-fold increase in the tip/cell body ratio of mChr-MORN4 in the presence, compared to the absence, of GFP-MYO3AΔK (Fig 5J), (mChr-MORN4 expressed with MYO3AΔK- 0.905 ± 0.071, mChr-MORN4 expressed with MYO3BΔK.3THDII- 0.369 ± 0.044, mChr-MORN4 only- 0.267 ± 0.011). These results show that mchr-MORN4 is more concentrated at filopodia tips relative to the cytoplasm in cells expressing GFP-MYO3AΔK. In contrast, MYO3BΔK.3THDII expression did not appreciably alter MORN4 distribution.

Our analysis (Fig 5J) also revealed an increased GFP-MYO3AΔK tip localization when GFP-MYO3AΔK was co-expressed with mChr-MORN4 as compared to that in the absence of mChr-MORN4 (GFP-MYO3AΔK expressed with mChr-MORN4- 3.59 ± 0.302, GFP-MYO3AΔK only- 1.78 ± 0.113). We speculate that when MYO3A is co-expressed with MORN4, the MYO3A-MORN4 complex gets tethered at the filopodia tips in a MORN4 dependent manner.

**Fig 6:**
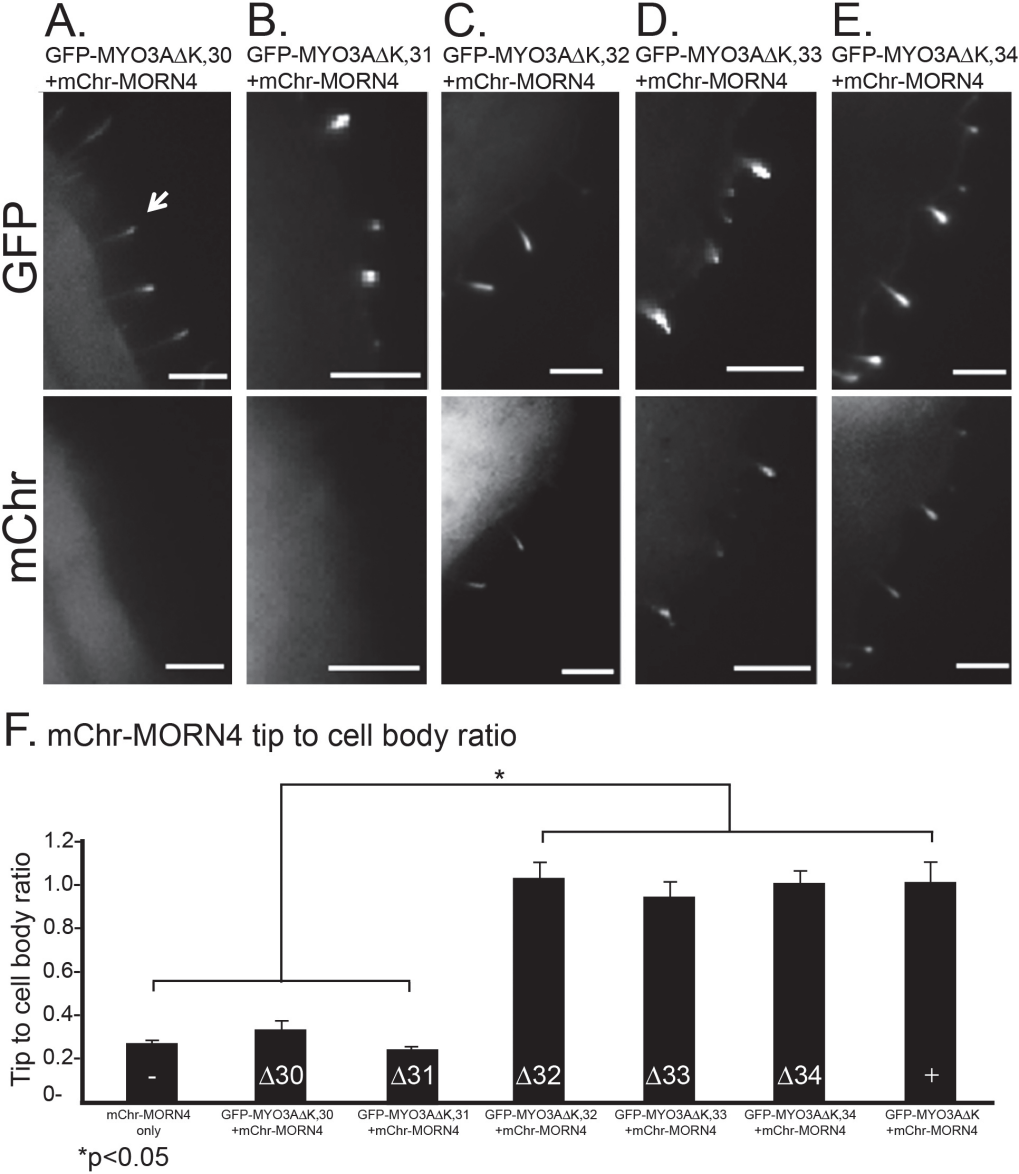
MYO3A tail exons 30 and 31 are required for localization of MORN4 to filopodial tips. (A-E) Representative COS7 cell images of mChr-MORN4 co-transfected with GFP-labeled MYO3A tail deletion constructs. The upper panel shows localization of each of the GFP-MYO3A tail deletion constructs and lower panel shows mChr-MORN4 localization. Scale bars, 5 μm. (F) Graph summarizing the tip to cell body ratio analysis of mChr-MORN4 cellular localization in presence of GFP-MYO3A constructs. When co-transfected with GFP-MYO3A constructs lacking tail exon 30 (p<0.0001) (n = 85 filopodia from 15 cells) and exon 31 (p<0.0001) (n = 65 filopodia from 13 cells) respectively, mChr-MORN4 has low tip to cell body ratio, while GFP-MYO3AΔK constructs lacking exon 32–34 respectively (GFP-MYO3AΔK,32 + mChr-MORN4: n = 57 filopodia from 12 cells; GFP-MYO3AΔK,33 + mChr-MORN4: n = 54 filopodia from 10 cells; GFP-MYO3AΔK,34 + mChr-MORN4: n = 96 filopodia from 11 cells) support mChr-MORN4 localization at the filopodial tips. Scale bars, 5 μm.

To map the MORN4 binding site within the MYO3A tail, we used derivatives of GFP-MYO3AΔK, in which one of the 6 exons (exons 30–35) encoding its tail domain were deleted. Exon 35 contains THDII, which is required for filopodia tip localization, and thus could not be examined. Each of the other MYO3A exon deletion constructs were capable of localizing to the filopodia tips when transfected into COS-7 cells. We found that the deletion of exons 30 and 31 prevented colocalization of MYO3A and MORN4 at the protrusion tips (Fig 6A and 6B). Deletion of exons 32, 33, and 34 did not prevent colocalization (Fig 6C–6E). We quantified the distribution of mChr-MORN4 in these cells by examining the filopodia tip/cell body ratio as done earlier [19]. The results (Fig 6F) indicate that deletion of MYO3A exons 30 or 31 result in a statistically significant reduction of MORN4 localization to filopodia tips relative to other MYO3A tail exons. This level of MORN4 localization is similar to that observed in cells lacking MYO3A, indicating that the sequence coded by MYO3A tail exons 30 and 31 are essential for MORN4 binding.

We conclude that MORN4 interacts to MYO3A but not MYO3B and that MORN4 may form a link between the plasma membrane and MYO3A leading to an increased MYO3A tip localization. On the basis of the GST pulldown assay and MORN4-MYO3A tail deletion constructs coexpression results it is likely that the direct MORN4 binding site is within carboxy tail sequences found in MYO3A but absent in MYO3B.

### Sequence Conservation within the RTP and MORN4 binding regions

**Fig 7:**
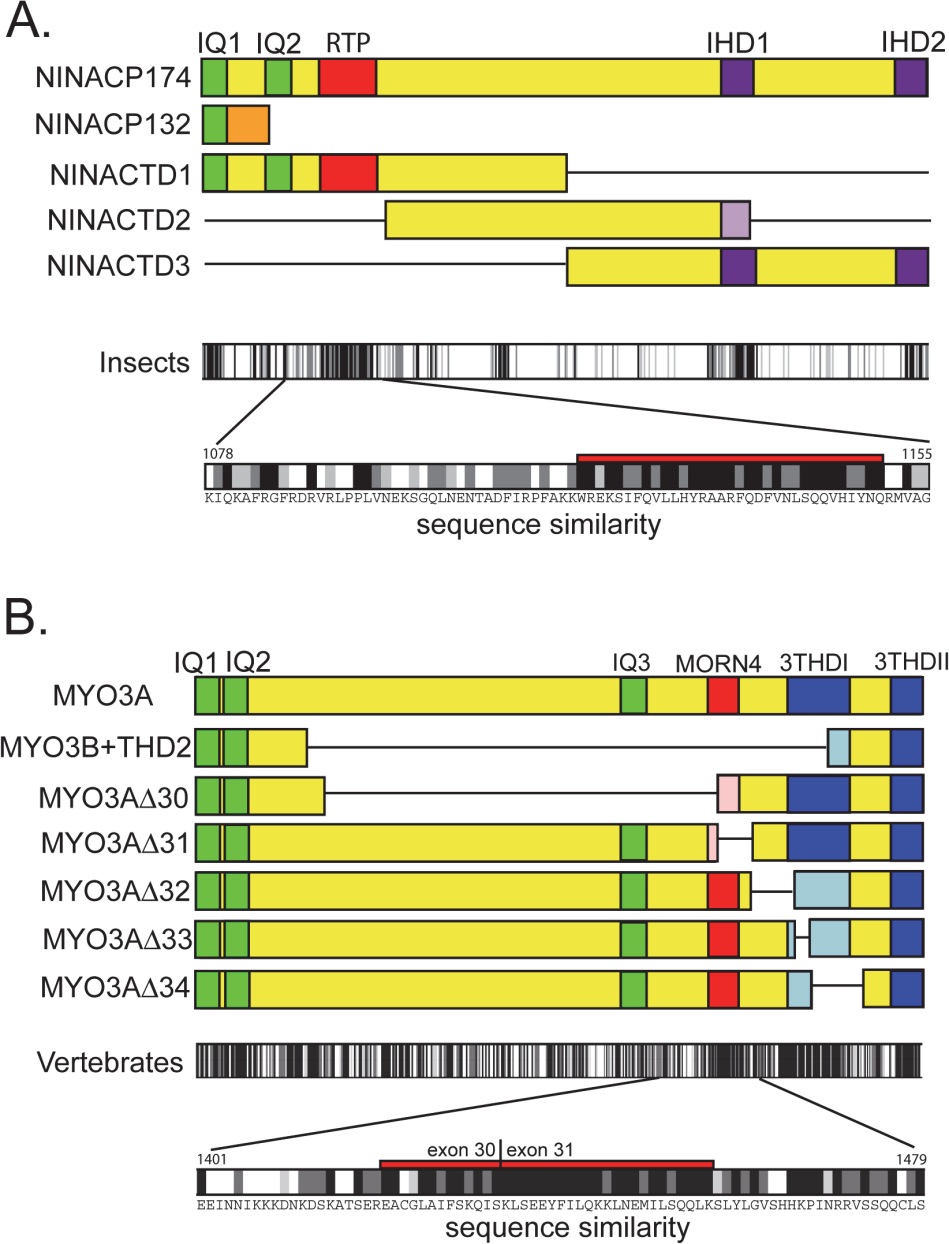
Identified and proposed motifs within the tail domain of *Drosophila* NINACp174 and human MYO3A. (A) The top diagram shows recognized and proposed tail motifs of NINACp174: IQ1 and IQ2 (green), IHD1 and IDH2 (invertebrate homology domains, based on high sequence identity, purple), and the RTP/NINAC interaction domain (red). Displayed below this diagram are the modifications to the tail structure for NINACp132 and deletions of NINACp174 that were used to map the RTP interaction domain. The lower part of the figure summarizes amino acid sequence identities of the *Drosophila* NINACp174 with MYO3 proteins of other invertebrate species to highlight the level of sequence identity within the RTP interaction domain. The invertebrate species used for the analysis were *Drosophila melanogaster*, *Drosophila yakuba*, the mosquito species *Aedes aegypti* and *Anopheles gambiae*, and the honeybee *Apis mellifera*. Identical amino acids are shown as black bars and functionally similar amino acids as two shades of grey bars as specified by Clustalx parameters [22]. At bottom, the region containing the proposed RTP binding site is expanded to show the amino acid sequence. (B) The top diagram shows recognized and proposed tail motifs of human MYO3A: IQ1, IQ2 and IQ3 (green), 3THDI and 3THDII (tail homology domains, blue), and the MORN4/MYO3A interaction domain (red). Displayed below this diagram are the modifications to the tail structure (modified MYO3B tail and deletions of MYO3A) that were used to map of the MORN4 interaction domain. The lower part of the figure summarizes amino acid sequence identities of human MYO3A with MYO3 proteins of other vertebrate species (chimpanzee, mouse, cow, chicken) highlighting the level of sequence identity within the proposed MORN4 interaction domain. Identical and functionally similar amino acids are coded as in (A). At bottom, the region containing the proposed MORN binding site is expanded to show the amino acid sequence within the MORN4 interaction domain.

Functional MYO3 tail domains were originally identified on the basis of sequence similarities in divergent species and subsequently shown to promote binding of specific proteins [5,20,21]. Similarly, because RTP is widely expressed in invertebrates, we asked whether the RTP binding site in NINAC was a conserved motif. Fig 7A shows the location of the RTP binding region and the IQ domains found within the NINACp174 tail. The sequence similarities of the insect NINACp174 tails we examined are indicated in the top bar graph of Fig 7A. The amino acid sequence within this region is shown in the second bar graph. Additional regions showing high conservation include the calmodulin binding sites (IQ1, IQ2) and two regions that correspond spatially to the 3THDI and 3THDII regions identified from comparative studies of vertebrate MYO3 sequences [5]. We refer to these homology domains as Invertebrate Homology Domain 1 and 2 (IHD1, and 2). We carried out a similar comparative analysis seeking a MORN4 binding site within the MYO3A tail. Fig 7B shows the exon deletions used to establish that both exons 30 and 31 were required for MORN4 binding. The bar graphs below these constructs identify a highly conserved sequence spanning the exon 30/31 boundary. Thus, this comparative approach identified a putative invertebrate RTP binding domain and a putative vertebrate MORN4 binding domain. However, we find little sequence identity between the invertebrate and vertebrate domains, and functional tests will be necessary to establish their role in both systems.

## Discussion

Prior studies showed that a photoreceptor specific myosin, NINAC, interacts with and stabilizes the small phosphoprotein RTP [8]. Here we extend this analysis to show that RTP, and the vertebrate ortholog MORN4, are novel myosin-binding proteins. We mapped the binding sites of RTP and MORN4 to the tail domains of NINAC and MYO3A, respectively. The carboxy tail domains of myosin family members possess the greatest sequence divergence and play key roles in their cellular control [3,23]. Consistent with the notion that these proteins act to regulate the behavior of these myosins, we show that the binding of RTP alters NINAC behavior, specifically by preventing formation of NINAC puncta.

### The proximal region of the NINACp174 tail contains the RTP binding site

RTP stability is dependent on NINACp174, and not NINACp132, suggesting that the RTP binding domain was contained in the carboxy tail region unique to the longer NINACp174 isoform. We constructed and expressed four NINACp174 tail regions in photoreceptor cells to delimit this RTP interaction domain. One construct represented the entire NINACp174 tail sequence, and three smaller constructs subdivided the tail into four regions. These NINAC fragments could be expressed in photoreceptor cells but did not traffic to the rhabdomeres.

The smallest NINAC fragment that stabilized and co-localized with RTP was NINACTD1. This fragment contains two IQ calmodulin binding motifs. The region unique to NINACTD1, that is not included in the NINACp132 tail or in NINACTD2, is a sequence that also contains IQ2. It is notable that this region is conserved in other invertebrate NINAC orthologs. A comparison of 5 arthropods shows a conserved segment on the carboxyl side of the IQ2 motif. The proximity of the RTP binding site to the unique IQ2 domain of NINACp174 suggests that the interplay between RTP and calmodulin may create an important control module for NINACp174 function. The *Drosophila* model provides excellent experimental approaches to further investigate these processes.

### Properties of the RTP/NINAC complex

A distinctive property of RTP is that its stability in photoreceptors is dependent on the presence of NINACP174. Ectopic expression of the RTP/NINAC complex in the neurons of the mushroom bodies and salivary glands allowed us to further characterize the requirements for RTP stabilization. NINAC-dependent RTP stability was maintained in both the neuronal cell types of the mushroom bodies, and the secretory cells of the salivary glands.

In both mushroom body neurons and salivary gland cells, we observed that RTP modifies NINAC behavior. In the absence of RTP, NINAC formed punctate structures near or within the cell nuclei. Co-expression of RTP with NINAC prevents the formation of punctate structures, with NINAC and RTP being co-localized and broadly distributed in the cells.

### The vertebrate orthologs MYO3A and MORN4 are also protein partners

The characterization of RTP/NINAC interactions led us to ask if a similar situation exists for the vertebrate orthologs. We used an *in vitro* protein affinity pull down assay to show that MORN4 directly binds to the tail domain of MYO3A. Further, *in vivo*, MORN4 is a cytoplasmic protein in the absence of MYO3A, but is colocalized with MYO3A at filopodial tips in the presence of MYO3A. The closely related protein MYO3B does not bring MORN4 to the filopodia tips, showing the importance of MYO3A specific motifs in promoting this interaction. Deletion constructs of the MYO3A tail show that both exons 30 and 31 are necessary for MORN4 tip localization, suggesting that MYO3A binds MORN4 and transports it to the filopodia tips. The amino acid sequence spanning the boundary of these two exons is highly conserved in vertebrate MYO3 orthologs, which may be a consequence of its role as a MORN4 binding domain.

The analysis revealed some key differences between the MYO3A/MORN4 interaction in vertebrate systems and the NINAC/RTP interaction in *Drosophila*. For example, RTP stability is dependent on NINAC, but MORN4 stability in COS-7 cells is not dependent on MYO3A. In *Drosophila* photoreceptors, degradation of excess RTP may provide a regulatory mechanism that is not in play in the vertebrate system. Another difference between systems involves the behavior of NINACp174 and MYO3A in the absence of their RTP/MORN4 binding partners. NINAC forms discrete puncta when expressed on its own in *Drosophila* neurons or salivary gland cells, but MYO3A localization in COS-7 cells is not dependent on MORN4. Interestingly, we observed an enhanced MYO3A filopodia tip localization in the presence of MORN4. Based on the previous reports suggesting that MORN repeat containing proteins act by linking proteins to the plasma membrane [10], we propose that MORN4 stabilizes MYO3A at the filopodia tips by tethering it to the membrane. The MYO3A anchoring affect in the presence of MORN4 may be a crucial aspect of MYO3A dependent actin bundle stabilization in vertebrate photoreceptor calycal processes where MYO3A is expressed [5]. In *Drosophila*, NINAC is only found in photoreceptors, and the localization is not dependent on RTP. In contrast, vertebrates express MYO3A in many tissues [5]. Thus multiple control elements are likely, and all may not be shared, in the invertebrate and vertebrate systems.

Here we have identified RTP and MORN4 as binding partners and a potential control element of NINACp174 and MYO3A. In both cases, binding occurs within the tail domain, the region associated with control of myosin motor cellular localization. RTP and MORN4 possess a tandem set of 4 MORN repeats, a motif found in other proteins and implicated in protein-protein interactions and protein binding to membranes [10,11]. These considerations show that RTP/MORN4 serve as adaptor proteins, binding to NINACp174/MYO3A. Their binding may enhance the association of the complex with membranes, or facilitate association with scaffolding and actin-based structures.
